# CaRinDB: an integrated database of common cancer mutations and residue interaction network parameters

**DOI:** 10.1093/bioadv/vbaf313

**Published:** 2026-01-25

**Authors:** Daniela Coelho Batista Guedes Pereira, João Vitor Ferreira Cavalcante, Laise Florentino Cavalcanti, Raul Maia Falcão, Jorge Estefano Santana de Souza, Rodrigo Juliani Siqueira Dalmolin, Thaís Gaudencio do Rêgo, Serghei Mangul, Gustavo Antônio de Souza, Patrick Terrematte, João Paulo Matos Santos Lima

**Affiliations:** Bioinformatics Multidisciplinary Environment (BioME), Digital Metropolis Institute (IMD), Universidade Federal do Rio Grande do Norte (UFRN), Natal, RN 59078-900, Brazil; Centro de Informática, Universidade Federal da Paraíba (UFPB), João Pessoa, PB 58058-600, Brazil; Bioinformatics Multidisciplinary Environment (BioME), Digital Metropolis Institute (IMD), Universidade Federal do Rio Grande do Norte (UFRN), Natal, RN 59078-900, Brazil; Bioinformatics Multidisciplinary Environment (BioME), Digital Metropolis Institute (IMD), Universidade Federal do Rio Grande do Norte (UFRN), Natal, RN 59078-900, Brazil; Bioinformatics Multidisciplinary Environment (BioME), Digital Metropolis Institute (IMD), Universidade Federal do Rio Grande do Norte (UFRN), Natal, RN 59078-900, Brazil; Bioinformatics Multidisciplinary Environment (BioME), Digital Metropolis Institute (IMD), Universidade Federal do Rio Grande do Norte (UFRN), Natal, RN 59078-900, Brazil; Bioinformatics Multidisciplinary Environment (BioME), Digital Metropolis Institute (IMD), Universidade Federal do Rio Grande do Norte (UFRN), Natal, RN 59078-900, Brazil; Centro de Informática, Universidade Federal da Paraíba (UFPB), João Pessoa, PB 58058-600, Brazil; Department of Clinical Pharmacy, USC Alfred E. Mann School of Pharmacy and Pharmaceutical Sciences, University of Southern California, Los Angeles, CA 90033, United States; Sage Bionetworks, Seattle, WA 98121, United States; Department of Biological and Morphofunctional Sciences, College of Medicine and Biological Sciences, Stefan cel Mare University of Suceava, Suceava 720229, Romania; Department of Computers, Informatics, and Microelectronics, Technical University of Moldova, Chisinau 2045, Moldova; Bioinformatics Multidisciplinary Environment (BioME), Digital Metropolis Institute (IMD), Universidade Federal do Rio Grande do Norte (UFRN), Natal, RN 59078-900, Brazil; Bioinformatics Multidisciplinary Environment (BioME), Digital Metropolis Institute (IMD), Universidade Federal do Rio Grande do Norte (UFRN), Natal, RN 59078-900, Brazil; Bioinformatics Multidisciplinary Environment (BioME), Digital Metropolis Institute (IMD), Universidade Federal do Rio Grande do Norte (UFRN), Natal, RN 59078-900, Brazil

## Abstract

**Motivation:**

Predicting the impact of missense mutations on protein structure and function is a fundamental challenge for cancer research and clinical applications. Despite all the computational advances and, more recently, the use of artificial intelligence (AI), assessing the functional consequences of residue substitutions remains a challenging task. Proteins have complex three-dimensional structures, where the maintenance of their functionality depends on chemical interactions between amino acid residues. Single substitutions can affect these interactions, leading to more profound structural changes that are difficult to visualize.

**Results:**

Here, we present CaRinDB, a database that integrates cancer-associated missense mutation data, functional predictions, molecular features, allelic frequencies, and residue interaction network (RIN) parameters derived from Protein Data Bank structures and AlphaFold models. Users can access and explore variant information through an intuitive web portal, with custom plots and tables to visualize and analyze cancer-associated mutation data. CaRinDB is the first database that unites distinct annotation features of cancer-associated mutations and their structural impacts, utilizing RINs graph parameters and a source of compiled and processed data for the development of AI tools.

**Availability and implementation:**

CaRinDB is freely available at https://bioinfo.imd.ufrn.br/CaRinDB/. The integrated development environment used was Jupyter notebooks, available on GitHub (https://github.com/evomol-lab/CaRinDB). CaRinDB web interface was implemented in R and Shiny.

## 1 Background and motivation

Predicting the impact of missense mutations on protein structure and function is essential for cancer research and clinical treatment (Cortés-Ciriano *et al.* 2022). Even with the recent application of artificial intelligence (AI), which has significantly expanded our capacity to obtain experimental-quality protein structure models (Aithani *et al.* 2023), assessing the functional effects of point residue changes remains a challenging task (Shea *et al.* 2023). The correct fold of a protein depends on establishing several chemical interactions, and their maintenance is crucial to function (Aydınkal et al. 2019). Since a single fold can accommodate highly diverse sequences, the conservation of these interactions commonly exceeds residue conservation within the sequence. One straightforward way to evaluate a residue’s connectivity with other amino acids or ligands is by constructing residue interaction networks (RINs) (Conte *et al.* 2024), which provide additional parameters of the mutated residue’s structural context to aid in automated evaluations of functional impact.

A variety of tools have been developed to manipulate pre-computed residue interaction networks, including RING (Conte *et al.* 2024), a widely adopted platform for generating high-quality RINs at the PDB scale; the Protein Contacts Atlas (Kayikci *et al.* 2018), an interactive resource of non-covalent contacts spanning over 100 000 PDB crystal structures; psnGPCRdb (Felline *et al.* 2023), a domain-specific precomputed structure-network (PSN/RIN) database for GPCR structures (consensus and ligand-centric networks); and RINmaker (Spanò *et al.* 2023), a versatile tool enabling the determination and visualization of RINs that encompass all standard non-covalent interactions. While these resources have significantly advanced the field, CaRinDB distinguishes itself as an interactive database that integrates cancer mutations from The Cancer Genome Atlas (TCGA) with their effect predictions and features. These features include RINs graph-based parameters, calculated from human Protein Data Bank structures, and AlphaFoldDB computer-predicted models, adding another layer of structural information. From CaRinDB, these distinct pieces of information can be retrieved and interactively plotted, providing additional features to assess the structural consequences of mutations. It also provides a structured data collection with residue connectivity parameters, allele frequencies, biological database integration, and functional predictions from distinct tools that can be used in AI training or machine learning applications.

## 2 Implementation

We retrieved cancer-associated SNPs from 33 tissues from the TCGA data repository (https://www.cancer.gov/tcga). We then performed functional annotations using ANNOVAR (Wang *et al.* 2010) and SnpEff (Cingolani *et al.* 2012), and cross-referenced gene and protein accession numbers with the GenBank (NCBI) and UniProt (uniprot.org) databases. From this latter, we retrieved the associated structures from PDB (rcsb.org) and AlphaFold Protein Structure Database (alphafold.ebi.ac.uk). The mmcif structure files were used as input for the RINs construction using RING 2.0 (Piovesan *et al.* 2016). We also retrieved additional annotations from ClinVar (https://www.ncbi.nlm.nih.gov/clinvar/) and AlphaMissense (Cheng *et al.* 2023). CaRinDB includes two datasets accessible through distinct tabs: CaRinDB, a set of 26 893 variants mapped to X-ray crystallography-resolved protein structures (resolution <3.0 Å) from the PDB, and CaRinDB::AlphaFold, a set of 201 447 variants merged with AlphaFold protein structures, with a mean global predicted local distance difference test score > 70. The first comprises experimental structures, and the other expands mutation’s structural coverage using computationally predicted models.

Additionally, for residues in wild-type protein structures, we incorporated RINs’ attributes, such as non-covalent interactions at the atomic level in protein structures, accounting for interaction types between amino acids and between amino acids and ligands. Furthermore, we performed graph-based calculations, including the clustering coefficient (measuring the tendency of nodes to cluster based on neighboring node clustering), node centrality (betweenness weighted, based on the number of shortest paths passing through a node), and the number of triangles formed by a node with other interacting residues (Supplementary Material, available as supplementary data at *Bioinformatics Advances* online). All the data preprocessing and engineering were implemented using the Python libraries Pandas and Numpy. The integrated development environment used was Jupyter notebooks, available on GitHub (https://github.com/evomol-lab/CaRinDB). CaRinDB web interface was implemented in R and Shiny (Supplementary Material 1, available as supplementary data at *Bioinformatics Advances* online) and is available at https://bioinfo.imd.ufrn.br/CaRinDB/.

## 3 Usage and data retrieving

**Figure 1:**
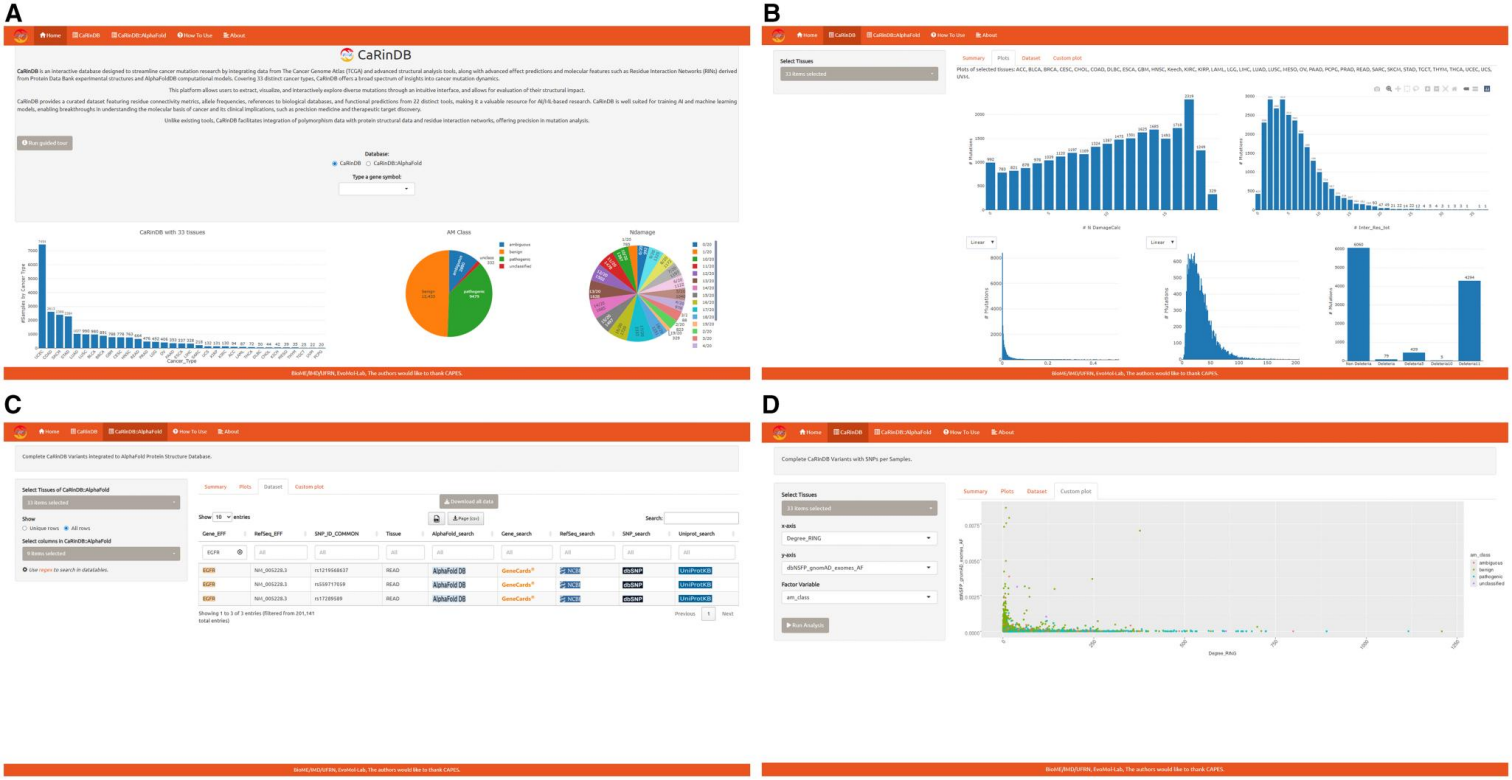
Overview of the CaRinDB database. (A) Main page. (B) RINs’ parameters plots. (C) Mutation data table. (D) Custom plot example.

First access to CaRinDB provides a brief database description and graphs showing general statistics of both datasets. The main graph displays the number of mutations categorized by type of cancer. At the same time, the pie chart illustrates the percentage of mutations classified according to the AlphaMissense class and the NDamage parameter, which represents the number of computational predictors that consider a particular mutation deleterious (Nascimento *et al.* 2020) (Fig. 1A). Users can also search the database using specific Gene Symbols. At the top of the page are tabs for individual access to the datasets, a “how-to-use” guide listing the data dictionary, and other CaRinDB functions, such as selecting the type of cancer, retrieving the data table, and creating customized plots.

Upon selecting a tab from one of the datasets, a descriptive and graphical summary of all columns, including information for all 33 types of cancer, is presented. On the right, the user can select one or more specific types for analysis. By clicking on Plots, users can visualize a set of graphs related to key database features. Based on their predictions, we separated the deleterious mutations into four classes (Supplementary Material—Fig. 1, available as supplementary data at *Bioinformatics Advances* online). In the same tab, another plot displays the number of mutations versus the B-factor or pLDDT of the residue present in the structure or model of the wild-type proteins from the PDB or AlphaFold sets, respectively. RINs’ parameters for that sequence site (wild-type residue) are also shown (Fig. 1B).

The next tab is a web visualization of the table with the selected mutation data (Fig. 1C). Users can choose the fields/columns and types of cancer from the menu on the right. The table presents direct links to other information databases. Specific buttons allow the complete download of all data in tabular files or only with the user-selected columns. In the last tab, the user can explore the data in customized plots for specific or all types of cancer (Fig. 1D). The user can determine the plot’s structure according to their parameter selection, as well as the x-axis, y-axis, and factor variable.

Below, we provide two examples that illustrate the usefulness of the data integration performed by CaRinDB. The first step is to examine mutations in the EGFR gene and the corresponding connectivity characteristics of its residues in the wild-type protein.

By choosing one of the datasets (PDB or AlphaFold) and keeping all 33 types of cancer selected, the user can go to the Dataset tab and insert “EGFR” in the search field on top of the Gene_EFF column (corresponding to the Gene Symbols used in TCGA), thus obtaining the table with all mutations in this gene in all types of cancer, as shown in Fig. 1C. The user can also restrict the search by allelic frequency obtained from the gnomAD database or limit it to deleterious mutations. In this last classification, the user can select the Deleteria11 class, which represents the decision tree used for the construction of the database (Supplementary Material, available as supplementary data at *Bioinformatics Advances* online) and which contains most of the pathogenic mutations classified by AlphaMissense, or can select between one or more results of the 21 predictors, including AlphaMissense’s prediction parameters (Cheng *et al.* 2023).

CaRinDB data integration also allows for specific questions about cancer-associated mutations, such as: Are there mutations in sites that are poorly connected but are still harmful or deleterious? What are the RIN parameters of the protein sites where mutations calculated as benign by AlphaMissense are present? What is the functional effect prediction from the different computational predictors? Which of the residue interaction network parameters are most associated with pathogenicity? All these questions can be explored by type of cancer using the custom plot construction function of CaRinDB. Figure 1D illustrates the customized graph generated from the analysis of the degree of connectivity (x-axis) versus the mutation frequency retrieved from the gnomAD database and categorized by color according to the classification obtained by AlphaMissense for all types of cancer included in CaRinDB. This graph indicates that the degree of connectivity of the residue is an important feature when considering the functional effect of a mutation. In fact, for both databases, a general trend is observed, where higher connectivity residue values have an increased proportion of probable pathogenic mutations, as annotated by AlphaMissense (Supplementary Material—Fig. 2, available as supplementary data at *Bioinformatics Advances* online).

We will continually update CaRinDB, and users can utilize Jupyter/Colab notebooks to customize their database or dataset. CaRinDB is a valuable tool for exploring the relationship between the characteristics of cancer-associated mutations and the RINs’ parameters. It quickly integrates polymorphism data, along with its functional and structural effects, into a structured database ready for AI applications.

## Supplementary Material

vbaf313_Supplementary_Data

## Data Availability

CaRinDB is freely available at https://bioinfo.imd.ufrn.br/CaRinDB/.
