# Supplementary material for: CaRinDB: an integrated database of common cancer mutations and residue interaction network parameters
Source: Bioinform Adv. 2026 Jan 25;6(1):vbaf313. doi: 10.1093/bioadv/vbaf313 (PMC12872580; doi:10.1093/bioadv/vbaf313)
Supplement: vbaf313_Supplementary_Data [file vbaf313_supplementary_data.zip › ID__BIOADV-2025-263_-_Supplementary_Material_-_new.docx]

**ADDITIONAL METHODS**

To construct the Residue Interaction Networks (RINs), we used the RING 2.0 program (Piovesan et al., 2016) available at <http://old.protein.bio.unipd.it/ring/>. We used as input mmcif files wild-type x-rays structures (resolution <3.0 Å) from PDB and model files modeled with AlphaFold2.0, available in AlphaFold Structure Database (<https://alphafold.ebi.ac.uk/>), with a mean global predicted local distance difference test, pLDDT_global (the average of all the residues pLDDT values from a protein model) >70. RING parameters were: Nodes closest, Thresholds strict; Edges All.

CaRinDB web interface was implemented in R (v. 4.3.1) with the Shiny (v. 1.8.1.1) and shinyWidgets (v. 0.8.6) packages. The platform utilizes the Plotly R package to generate on-demand plots, and dynamic tables are displayed using the DT package (version 0.33), which provides an R interface to the JavaScript library DataTables. We also used the R package memoise (v. 2.0.1) to optimize the application and cache memory. All the scripts are on [https://github.com/evoMOL-La](https://github.com/evoMOL-Lab)b. The web interface is available at [https://bioinfo.imd.ufrn.br/CaRinDB](https://bioinfo.imd.ufrn.br/CaRinDB/)/.

To calculate the clustering coefficient, we use the following equation:


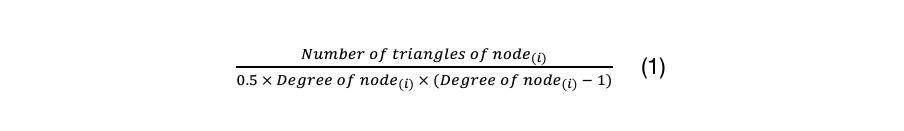


The estimated clustering coefficient ranges from 0 to 1, where 1 is the maximum possible number of connections.

To calculate the number of triangles formed by a node with other interacting residues, we use the function count_triangles of the R package igraph (Csardi and Nepusz, 2006 - <http://igraph.org/>).

To calculate the node centrality (in-betweenness weighted, based on the number of shortest paths passing through a node), we use the betweenness function of igraph (Csardi and Nepusz, 2006 - http://igraph.org/). The results range from 0 to 1. Therefore, a value of 0.1 means that 10% of the shortest pathways of the network pass through that node.

**Decision Tree of DeleteriaN labels**

The Decision Tree presented (Supplementary Figure 1) describes how the DeleteriaN label (used to classify genetic variants as benign or pathogenic) was constructed. The classification begins with the evaluation of the predicted impact on protein function using three computational tools: SIFT, PolyPhen2_Dam (the Junction of the predictors PolyPhen2_HDIV and PolyPhen2_HVAR), and PROVEAN. If at least one tool predicts a harmful effect, the variant is subjected to additional analysis.

Next, the frequency of the variant in the general population, obtained from the gnomAD database, is considered. Variants with a frequency of less than 0.0001 are considered rare and proceed to the next step. At this stage, the potential damage to the protein caused by the variant is assessed using NdamageCalc (the number of predictors that classified this mutation as pathogenic). For each cutoff of 3, 5, 10, and 11, if the calculated damage is less than the respective cutoff value, the variant is classified as benign.

Finally, the frequency of the mutation in the specific genomic region is evaluated. A high frequency suggests a benign variant, while a low frequency indicates a pathogenic variant. This flowchart integrates several rules of evidence for classifying genetic variants, providing a systematic approach to variant interpretation.


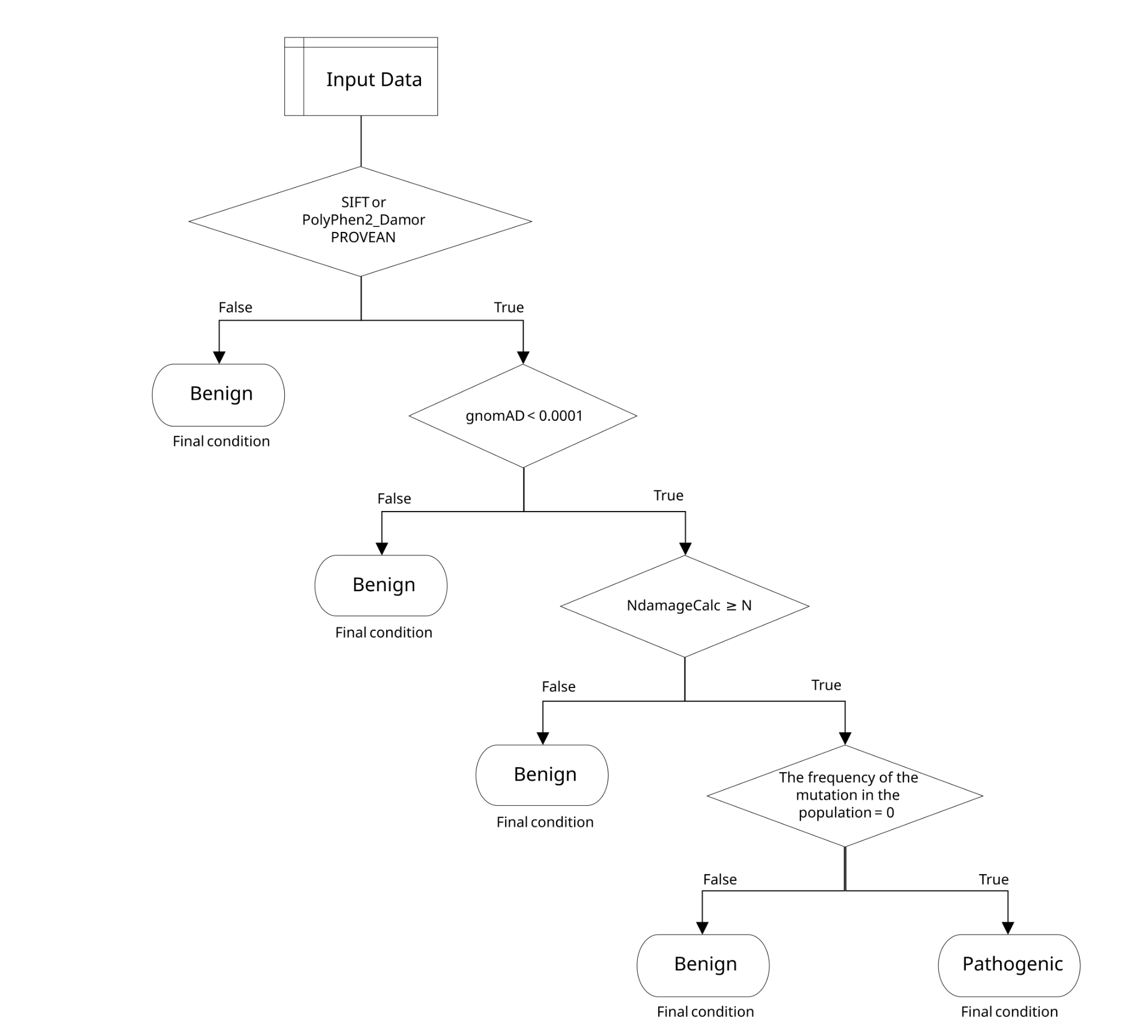


**Supplementary Figure 1**. Decision tree to classify mutations listed in the CaRinDB. NDamageCalc ≥ N stands for each cutoff value of 3, 5, 10, and 11.


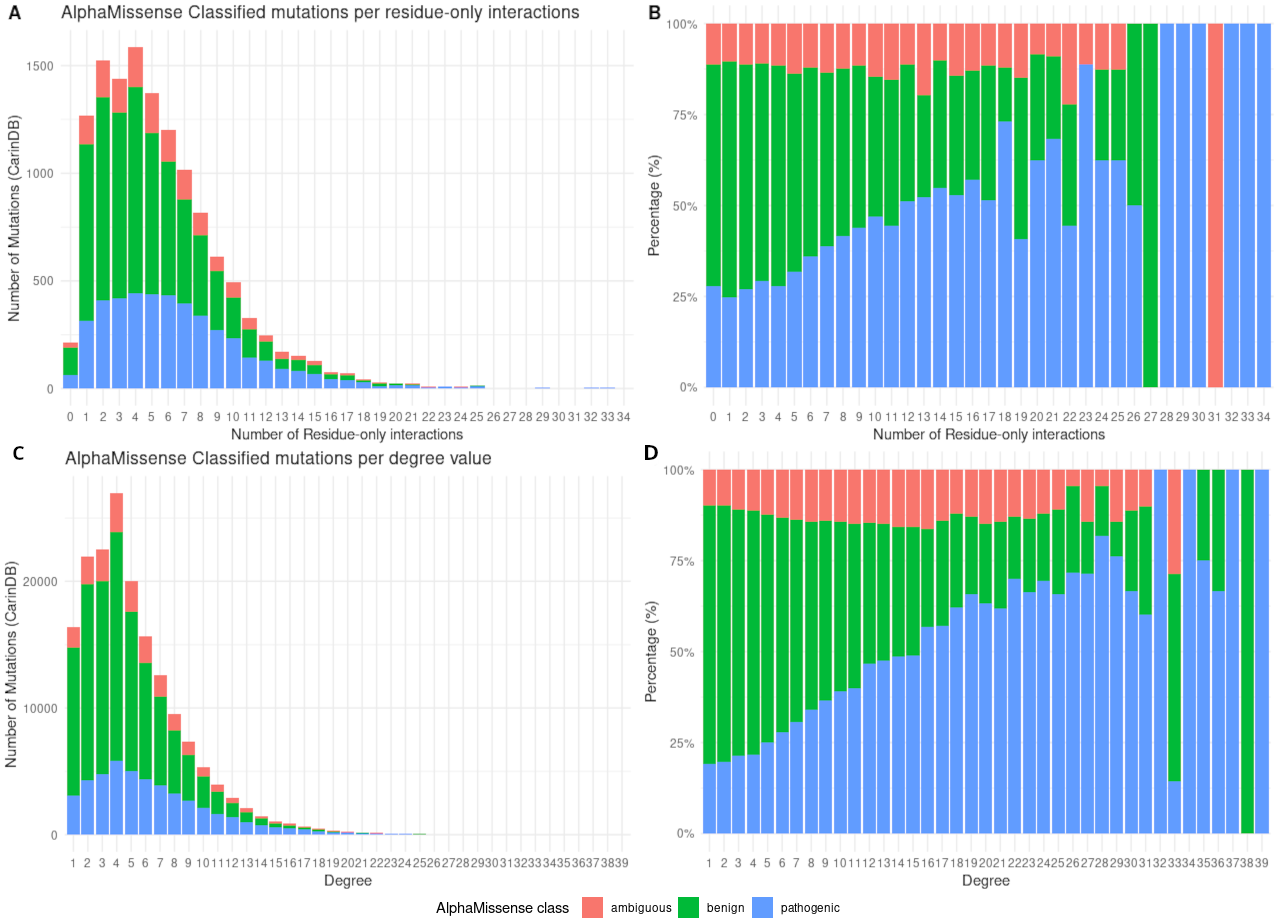


**Supplementary Figure 2**. Residues’ connectivity values classified by AlphaMissense predictions from CaRinDB (A and B) and CaRinDB::AlphaFold (C and D). Plots A and C y-axis represent the total number of mutations, and plots B and C y-axis represent the proportion of each type of mutation versus the number of residue-residue interactions (degree).

**REFERENCES**

Csardi G, Nepusz T (2006). “The igraph software package for complex network research”. InterJournal, Complex Systems, 1695.

Piovesan, D., Minervini, G., Tosatto, S. (2016). The RING 2.0 web server for high quality residue interaction networks. Nucl. Acids Res. 44(W1), W367-W374. https://dx.doi.org/10.1093/nar/gkw315.
