# Supplementary figures and images for: CaRinDB: an integrated database of common cancer mutations and residue interaction network parameters
Source: Bioinform Adv. 2026 Jan 25;6(1):vbaf313. doi: 10.1093/bioadv/vbaf313 (PMC12872580; doi:10.1093/bioadv/vbaf313)

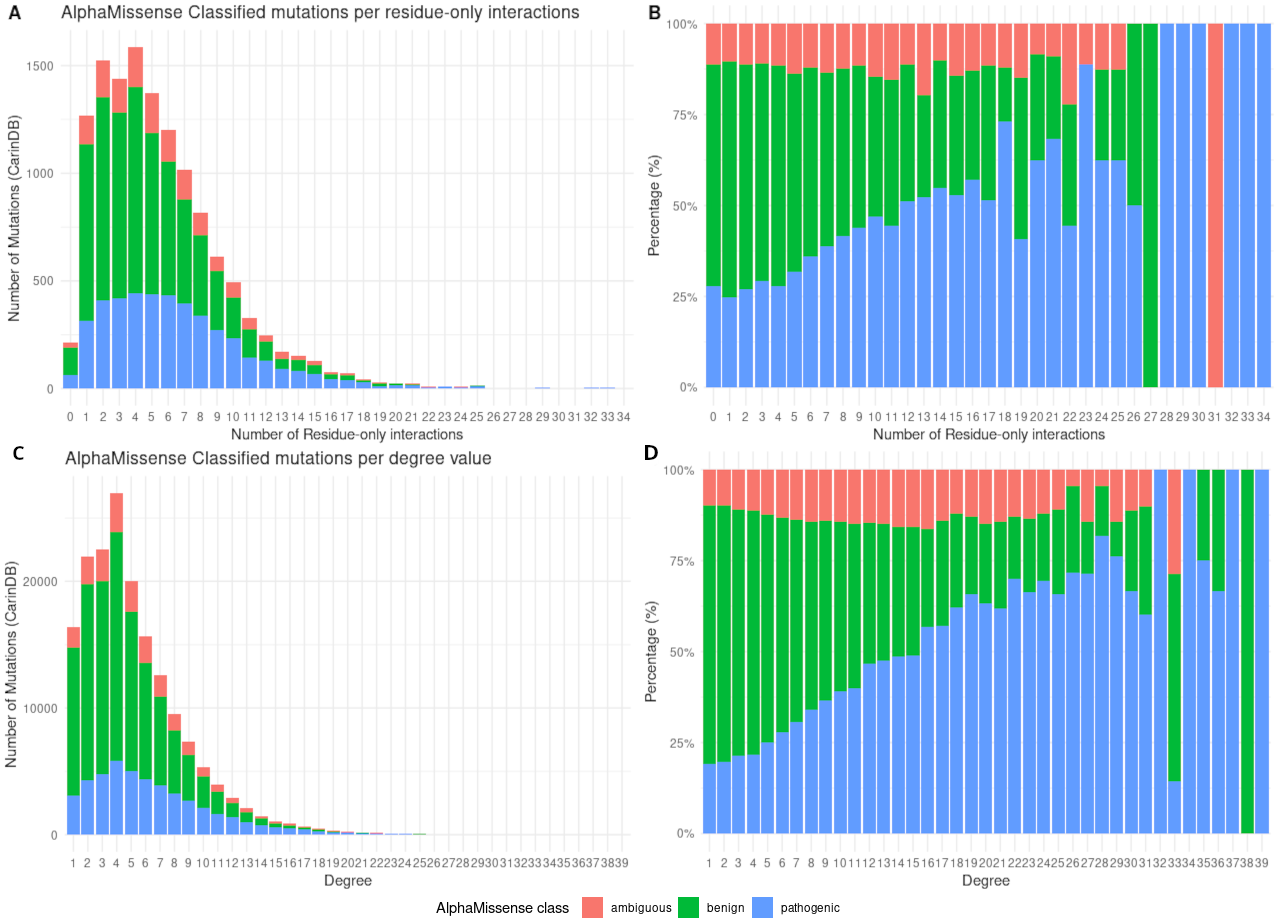

Supplement: vbaf313_Supplementary_Data [file vbaf313_supplementary_data.zip › Supplementary Figure 2.png]

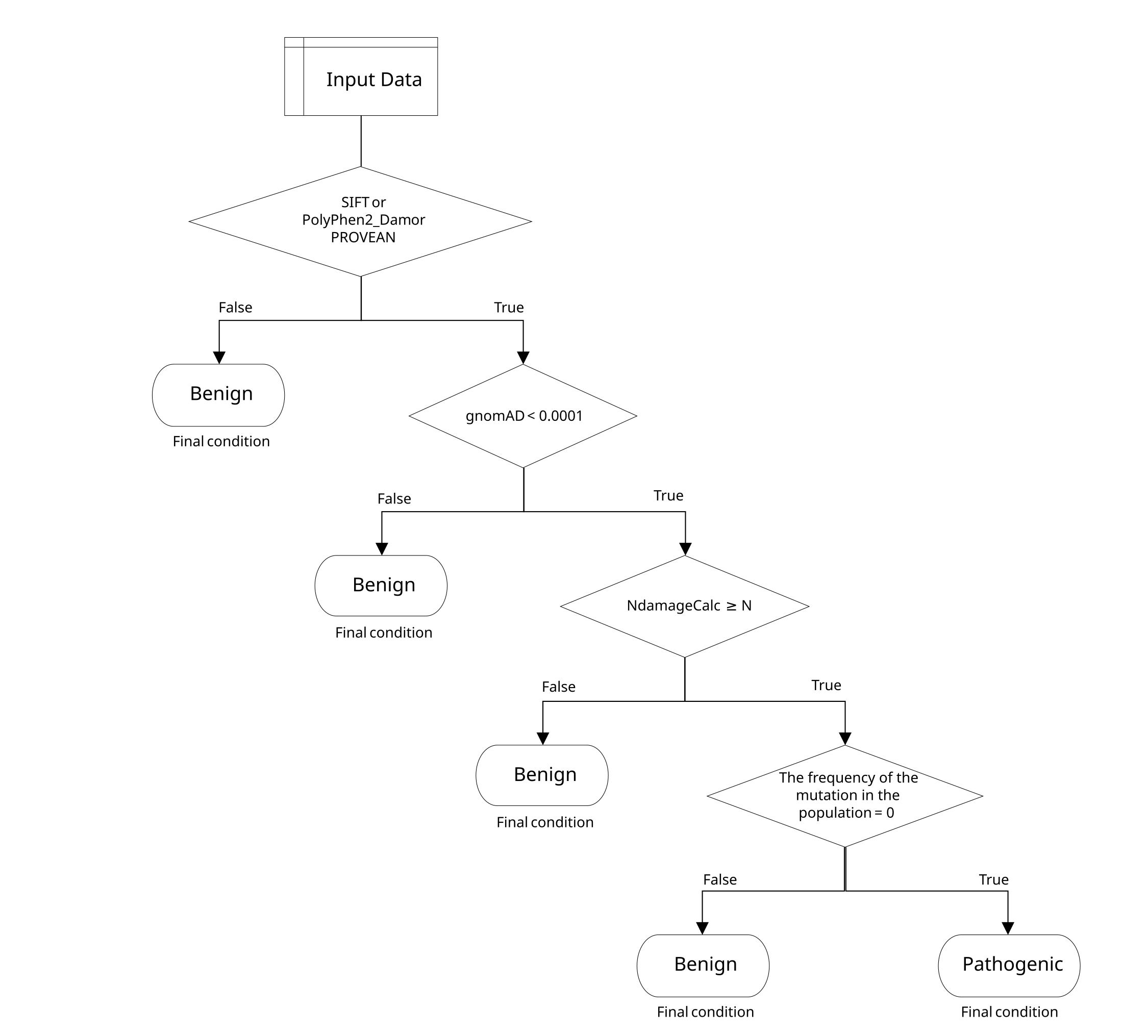

Supplement: vbaf313_Supplementary_Data [file vbaf313_supplementary_data.zip › SupplementaryFigure_1.png]
